# Supplementary material for: Unsupervised pretraining in biological neural networks
Source: Nature. 2025 Jun 18;644(8077):741–8. doi: 10.1038/s41586-025-09180-y (PMC12367527; doi:10.1038/s41586-025-09180-y)
Supplement: Supplementary file 1 — Reporting Summary [file 41586_2025_9180_MOESM1_ESM.pdf]

Corresponding author(s): Marius Pachitariu

Last updated by author(s): May 8, 2025

Reporting Summary

Nature Portfolio wishes to improve the reproducibility of the work that we publish. This form provides structure for consistency and transparency in reporting. For further information on Nature Portfolio policies, see our Editorial Policies and the Editorial Policy Checklist.

Statistics

For all statistical analyses, confirm that the following items are present in the figure legend, table legend, main text, or Methods section.

n/a	Confirmed	
☐	☒	The exact sample size (n) for each experimental group/condition, given as a discrete number and unit of measurement
☐	☒	A statement on whether measurements were taken from distinct samples or whether the same sample was measured repeatedly
☐	☒	The statistical test(s) used AND whether they are one- or two-sided<br>*Only common tests should be described solely by name; describe more complex techniques in the Methods section.*
☒	☐	A description of all covariates tested
☒	☐	A description of any assumptions or corrections, such as tests of normality and adjustment for multiple comparisons
☐	☒	A full description of the statistical parameters including central tendency (e.g. means) or other basic estimates (e.g. regression coefficient) AND variation (e.g. standard deviation) or associated estimates of uncertainty (e.g. confidence intervals)
☐	☒	For null hypothesis testing, the test statistic (e.g. F, t, r) with confidence intervals, effect sizes, degrees of freedom and P value noted<br>*Give P values as exact values whenever suitable.*
☒	☐	For Bayesian analysis, information on the choice of priors and Markov chain Monte Carlo settings
☒	☐	For hierarchical and complex designs, identification of the appropriate level for tests and full reporting of outcomes
☐	☒	Estimates of effect sizes (e.g. Cohen's d, Pearson's r), indicating how they were calculated

Our web collection on statistics for biologists contains articles on many of the points above.

Software and code

Policy information about availability of computer code

Data collection	Matlab 2022, psychtoolbox 3.0.19
Data analysis	python 3.12.8, numpy 2.0., scipy 1.14.1, matplotlib 3.10.0, suite2p 0.14.4, rastermap 0.1.3, suite2p 0.14.2

For manuscripts utilizing custom algorithms or software that are central to the research but not yet described in published literature, software must be made available to editors and reviewers. We strongly encourage code deposition in a community repository (e.g. GitHub). See the Nature Portfolio guidelines for submitting code & software for further information.

Data

Policy information about availability of data

All manuscripts must include a data availability statement. This statement should provide the following information, where applicable:

- Accession codes, unique identifiers, or web links for publicly available datasets
- A description of any restrictions on data availability
- For clinical datasets or third party data, please ensure that the statement adheres to our policy

Data is now available at https://doi.org/10.25378/janelia.28811129.v1
Accompanying code to reproduce analyses in the paper is available at https://github.com/MouseLand/zhong-et-al-2025.

Research involving human participants, their data, or biological material

Policy information about studies with human participants or human data. See also policy information about sex, gender (identity/presentation), and sexual orientation and race, ethnicity and racism.

Reporting on sex and gender	n/a
Reporting on race, ethnicity, or other socially relevant groupings	n/a
Population characteristics	n/a
Recruitment	n/a
Ethics oversight	n/a

Note that full information on the approval of the study protocol must also be provided in the manuscript.

Field-specific reporting

Please select the one below that is the best fit for your research. If you are not sure, read the appropriate sections before making your selection.

☒ Life sciences ☐ Behavioural & social sciences ☐ Ecological, evolutionary & environmental sciences

For a reference copy of the document with all sections, see nature.com/documents/nr-reporting-summary-flat.pdf

Life sciences study design

All studies must disclose on these points even when the disclosure is negative.

Sample size	No sample size calculation was performed. We used 3-9 mice for each group, which is common for neural recordings with behavior, and sufficient due to recording a very large number of neurons per mouse (~50,000). In cases where 3 mice were used (naive group), multiple sessions were repeated with independent stimulus sets and treated as different samples.
Data exclusions	No data was excluded.
Replication	Each mouse trained and recorded can be viewed as a replication of the experiment. Each mouse generated a dataset of about 50,000 neurons per session, and all the major results held in each mouse individually.
Randomization	Allocation was random.
Blinding	Investigators were not blinded, because the supervised cohort of mice had to be water restricted and trained to produce the behavioral choices.

Reporting for specific materials, systems and methods

We require information from authors about some types of materials, experimental systems and methods used in many studies. Here, indicate whether each material, system or method listed is relevant to your study. If you are not sure if a list item applies to your research, read the appropriate section before selecting a response.

Materials & experimental systems

n/a	Involved in the study	
☒	☐	Antibodies
☒	☐	Eukaryotic cell lines
☒	☐	Palaeontology and archaeology
☐	☒	Animals and other organisms
☒	☐	Clinical data
☒	☐	Dual use research of concern
☒	☐	Plants

Methods

n/a	Involved in the study	
☒	☐	ChIP-seq
☒	☐	Flow cytometry
☒	☐	MRI-based neuroimaging

Animals and other research organisms

Policy information about studies involving animals; ARRIVE guidelines recommended for reporting animal research, and Sex and Gender in Research

Laboratory animals	The study involved C57 mice as well as gcamp6s transgenic mice (tetO-gcamp6s x Camk2a-tTA), both male and female, at 2-11 months of age. The mice were housed in reversed light cycle in individually ventilated cages kept at 46-57% humidity and 21-22C.
Wild animals	No wild animals were used in this study.
Reporting on sex	Both female and male mice were used, but we could analyze data by subgroup because the experiments are labor-intensive and not enough animals were recorded.
Field-collected samples	No field samples were used in this study.
Ethics oversight	All experimental procedures were conducted according to IACUC, and received ethical approval from the IACUC board at HHMI Janelia Research Campus.

Note that full information on the approval of the study protocol must also be provided in the manuscript.

Plants

Seed stocks	N/A
Novel plant genotypes	N/A
Authentication	N/A
